# Hidden Beats: Detection of Subclinical Atrial Fibrillation Using Questionnaires in Young Adults and Its Cardiovascular Risk Profile

**DOI:** 10.14789/ejmj.JMJ25-0068-OA

**Published:** 2026-03-04

**Authors:** JUNAID AYAZ KHAN, SIDRA ANWAR, BEENISH ALI BAIG, OSAGIE JOY IGUODALA, SHINU PHILIP, KINZA RIAZ, HANIYYA HUSSAIN CP, SHAUMILE HASAN KHAN, QAISER KHAN, MISHA MOHAMMADI, MAKURIRA VUNYOROHWASHE

**Affiliations:** 1 Department of General Medicine, Sun Yat-sen Medical College, Guangzhou, China; 1 Department of General Medicine, Sun Yat-sen Medical College, Guangzhou, China; 2 Department of Cardiology, Shaikh Zayed Hospital, Lahore, Pakistan; 2 Department of Cardiology, Shaikh Zayed Hospital, Lahore, Pakistan; 3 Department of Medicine, CDA Hospital, Islamabad, Pakistan; 3 Department of Medicine, CDA Hospital, Islamabad, Pakistan; 4 Department of Internal Medicine, College of Medical Sciences, University of Benin, Benin City, Nigeria; 4 Department of Internal Medicine, College of Medical Sciences, University of Benin, Benin City, Nigeria; 5 Department of Medicine, Kharkiv National Medical University, Kharkiv, Ukraine; 5 Department of Medicine, Kharkiv National Medical University, Kharkiv, Ukraine; 6 Department of Internal Medicine, Birmingham Heartlands Hospital, Birmingham, United Kingdom; 6 Department of Internal Medicine, Birmingham Heartlands Hospital, Birmingham, United Kingdom; 7 Department of Medicine, Burjeel Holdings, Abu Dhabi, United Arab Emirates; 7 Department of Medicine, Burjeel Holdings, Abu Dhabi, United Arab Emirates; 8 Department of Medicine, FMH Medical College, Lahore, Pakistan; 8 Department of Medicine, FMH Medical College, Lahore, Pakistan; 9 Department of Cardiology, Hayatabad Medical Complex, Peshawar, Pakistan; 9 Department of Cardiology, Hayatabad Medical Complex, Peshawar, Pakistan; 10 Department of Public Health, York St John University, London, United Kingdom; 10 Department of Public Health, York St John University, London, United Kingdom; 11 Department of Acute Medicine, Royal Cornwall Hospital, Truro, United Kingdom; 11 Department of Acute Medicine, Royal Cornwall Hospital, Truro, United Kingdom

**Keywords:** subclinical atrial fibrillation, cardiovascular risk, quality of life, INTERHEART risk score

## Abstract

**Objectives:**

The objectives were to identify subclinical AF in young individuals and to examine its association with cardiovascular risk profile and quality of life (QoL) in a young adult cohort.

**Design:**

A cross-sectional survey was conducted with 403 adults aged 18-40 years in public and private hospitals.

**Methods:**

The INTERHEART Modifiable Risk Score (IHMRS), Atrial Fibrillation Effect on Quality-of-Life (AFEQT) questionnaire, and the Modified European Heart Rhythm Association (mEHRA) were used to collect data. The statistics were performed using descriptive statistics, Spearman's correlation, the Mann-Whitney U test, the Kruskal-Wallis test, and multiple linear regression in IBM SPSS 26.

**Results:**

Of the 403 respondents, the majority were male (85%) and aged 36-40 (30%). A stronger negative correlation with QoL (0.42, p < 0.001) and a weaker positive correlation with cardiovascular risk (0.13, p = 0.009) were observed between higher EHRA symptom severity and cardiovascular risk. There was also a negative correlation between QoL and IHMRS (r = -0.29, p  = < 0.001). Women presented with greater symptoms and low QoL (p < 0.001). The regression analysis showed that predictors of better IHMRS were age, male gender, higher EHRA scores, physical inactivity, and a family history of CVD. In contrast, better QoL was a protective factor (p < 0.01).

**Conclusions:**

Subclinical AF symptoms were highly correlated with increased cardiovascular risks and decreased QoL in young adults. Early detection of high-risk individuals, especially in settings with limited resources, could be achieved through simple questionnaire-based screening.

## Introduction

Atrial fibrillation (AF), the most prevalent sustained arrhythmia, afflicts more than 33 million individuals worldwide and is projected to double in prevalence by 2050, causing significant morbidity of stroke, heart failure, dementia and premature death^1, 2)^. Recent research has demonstrated that atrial fibrillation (AF) occurs on a susceptible background in which unmodifiable factors (age, sex, genetics) and modifiable risk factors (e.g., smoking, obesity, hypertension, diabetes, sleep apnea) have an impact^3)^.

AF frequently accompanies congestive heart failure (CHF), and the presence of atrial fibrosis and structural remodelling raises AF susceptibility. Both experimental and clinical studies support the roles of the angiotensin and transforming growth factor-β (TGF-β) pathways in this regard, suggesting potential therapeutic targets^4)^. Quality of life (QoL) is severely impaired in patients with atrial fibrillation (AF)disease compared to the general population^5)^.

The risk of stroke in atrial fibrillation (AF) varies markedly, with previous stroke/transient ischaemic attack (TIA), increasing age, hypertension, and diabetes being the most consistently identified independent predictors^6)^. AF is associated with a substantially increased risk of cardiovascular complications, stroke, renal disease and HF^7)^, and women are at greater risk than men for CVD, stroke and mortality^8)^.

Beyond cardiovascular conditions, AF frequently occurs in conjunction with noncardiac diseases, such as cancer, sepsis, chronic obstructive pulmonary disease (COPD), obstructive sleep apnea (OSA), and chronic kidney disease, that are mediated by standard pathophysiological mechanisms of inflammation and autonomic dysfunction^9)^. Traditional cardiovascular risk factors, such as hypertension and obesity, are associated with a higher risk of incident AF. At the same time, cholesterol and diastolic blood pressure are inconsistently or inversely associated with it, suggesting deviation from traditional CVD risk^10)^.

The study of the prevalence of subclinical AF among young adults and the relationship with cardiovascular risk factors can guide preventive measures and early interventions. Nonetheless, limited information is available on this group, particularly regarding questionnaire-based screening. The study aims to identify subclinical AF in young adults using a structured questionnaire and to define their cardiac risk profile, thereby providing better insight into the early diagnosis and prevention of AF-related complications.

### Novelty and study gap

Atrial fibrillation (AF) is usually a subject of research in older populations, whereas younger adults exhibiting subclinical forms of the condition receive little attention. Most previous studies have used costly or invasive detection methods, such as Holter monitoring, wearable devices, or echocardiography, which are often not feasible in resource-poor settings. As a result, there is no evidence available on the early detection of subclinical AF in young adults, nor its relationship with cardiovascular risk factors and quality of life (QoL).

This research fills a void amidst the different methodologies by systematically assessing the applicability of non-intrusive and straightforward questionnaires—the INTERHEART Modifiable Risk Score (IHMRS), the Atrial Fibrillation Effect on Quality-of-Life (AFEQT) questionnaire, and the Modified European Heart Rhythm Association (mEHRA) scale— for screening subclinical AF in the younger adults (18-40 years) category. Doing so through discerning the interrelations between AF symptoms, heart disease risk, and QoL, the research not only brings in a cost-efficient and user-friendly way for the early detection of such individuals who are prone to complications but also makes a fresh contribution to the AF study of young adults in resource-poor areas, thus securing a wide range of applicability in the health sector.

### Objectives

The main aim of the study was to approximate the prevalence of subclinical atrial fibrillation in young subjects with a standardised questionnaire. The secondary aims were to assess the cardiovascular risk profile of young adults with subclinical AF identified through questionnaire-based screening, to examine the relationships between demographic and lifestyle factors and AF symptoms, and to determine the usefulness of this method for early detection in adults.

## Materials and Methods

To identify subclinical AF and cardiovascular risk profiles in young adults, we conducted a cross-sectional observational study. To represent a diverse population from economic and cultural perspectives, participants were recruited from the inpatient and outpatient departments of hospitals (which serve both public and private patients). The instrument was a structured, interviewer-administered questionnaire that collected information at a single point in time on possible symptoms, risk indicators for AF, and lifestyle factors.

Trained research assistants visited the clinically stable patients to explain the study objectives and procedures. All respondents provided written or oral informed consent. The participants themselves completed the questionnaires or, if illiterate, with the assistance of a research team member. Accurate, culturally tailored and respectful data collection in the hospital environment ensured identification of young adults at risk for subclinical AF and associated CV complications.

### Recruitment & sample details

The prevalence (p) of subclinical atrial fibrillation (AF) in young adults was assumed to be 0.5, and the population was considered infinite because of the lack of local data on the pooled prevalence. The required sample size was calculated at the 95% confidence level (Z = 1.96) and a margin of error of 0.05^11)^. After these parameters, a sample size of 384 people was determined to be the minimum required. A convenience sampling technique was employed to recruit participants from the outpatient and inpatient departments of the selected hospitals.

Of the 450 individuals approached, 47 were not eligible or declined to participate. The other 403 participants provided written or oral informed consent, completed the structured questionnaire, and were subsequently included in the final analysis. Convenience sampling enabled the researcher to conduct recruitment efficiently and cost-effectively. Still, it can also provoke selection bias, reducing the possibility of generalising the results to the rest of the population.

### Eligibility criteria

The sample consisted of young adults aged 18 to 40 years who visited an outpatient or inpatient department of identified hospitals, were clinically stable, capable of making informed consent, and ready to complete the structured questionnaire. The study excluded individuals who had been previously diagnosed with atrial fibrillation or other significant arrhythmias, and those who had experienced a major cardiovascular event previously, like myocardial infarction or stroke, or were too critically ill or incapable of taking part in the study. This methodology helped recruit a representative sample of at-risk young adults, ensuring participant safety and the reliability of the information.

### Instruments and measures

A structured questionnaire was used to collect detailed data for this study. It was divided into three sections: demographic parameters and INTERHEART Modifiable Risk Score (MRS); the Atrial Fibrillation Effect on Quality of Life (AFEQT) questionnaire; and the modified EHRA (mEHRA) stage. All instruments were presented in the original English version, and no linguistic or cultural adaptation was made.

### Demographic information

The first part gathered demographic and clinical characteristics for subgroup analysis and evaluated the correlation with subclinical AF. Variables included age, sex, marital status, level of education, occupation and whether they lived in urban or rural areas. Clinical data included associated diseases and cardiovascular history.

### INTERHEART modifiable risk score (IHMRS)

All participants' cardiovascular risk was estimated using the INTERHEART Modifiable Risk Score (IHMRS), first presented by Yusuf and colleagues in the 2004 INTERHEART study and further validated in 2010. It evaluates the modifiable risk factors, including smoking, blood pressure, diabetes, body mass index (BMI), diet, physical activity, lifestyle stress and alcohol. The IHMRS has excellent predictive ability in different populations, including South Asians. It often has an AUC over 0.75, suggesting it is good at distinguishing risk between individuals. Given its validity and ease of use, IHMRS is a practical, worthwhile tool for estimating cardiovascular risk in the population^12)^.

### Atrial fibrillation effect on quality of life (AFEQT) questionnaire

Quality of life was assessed via the Atrial Fibrillation Effect on Quality of Life (AFEQT) questionnaire developed by Spertus et al. (2009). It is a 20-item patient-rated measure that includes the following subscales: symptoms, daily activities, treatment concerns, and treatment satisfaction. All responses are rated on a 7-point Likert scale with higher scores reflecting better QoL. The domain scores are then summed to yield a total score, on a 0-100 scale. The scale has demonstrated high internal consistency, as indicated by a Cronbach's alpha of 0.90 or higher. The AFEQT has also been used in the current study to detect small decrements in HRQOL among the youth with subclinical AF and to contribute data on relationships among atrial fibrillation (AF) symptoms, functional impairment and cardiovascular risk models^13)^.

### Modified european heart rhythm association (mEHRA) classification

To quantify the impact of atrial fibrillation (AF) symptoms, Young et al. adapted the European Heart Rhythm Association (EHRA) AF symptom classification system, known as the Modified mEHRA classification, developed by Wynn et al. in 2014. The tool classifies patients according to the extent to which the AF impacts their daily functions, with no symptoms (Class 1), to severe or disabling symptoms that have serious restrictions on normal functioning (Classes 2a, 2b, 3, and 4). Even though it lacks a numerical total score, each class symbolises a progressive increase in symptom severity, providing a simple, validated way to grade them consistently. Its application in this study enabled the standardised evaluation of symptom severity among young adults with subclinical or undiagnosed AF, thereby supporting the analysis of relationships between symptom burden and cardiovascular risk factors^14)^.

### Data collection process

Eligible participants were contacted upon arrival at the outpatient or inpatient departments of the selected hospitals. Only the members of the clinical stable population who met the inclusion criteria and gave informed consent were enrolled. The data were collected over six months, from March 2025 to August 2025. The questionnaire was structured and completed independently or with the help of research teams, depending on participants' literacy levels and preferences. Every answer was documented systematically to avoid any misinterpretation.

### Data analysis strategy

Data analysis was performed using IBM SPSS Statistics version 26 (IBM Corp., Armonk, NY, USA). Demographic and clinical characteristics were summarised using descriptive statistics (frequencies and percentages). Rank-order correlations were used to evaluate the associations among the EHRA, AFEQT, and IHMRS. These gender-based differences between the groups were compared using the Mann-Whitney U test, whereas age-group differences were analysed using the Kruskal-Wallis H test. A predictive model of INTERHEART Risk Scores was developed using the following predictors: demographic (age, gender, physical activity, family history of CVD) and clinical (EHRA, AFEQT) variables, via multiple linear regression. A p-value with less than 0.05 was regarded as statistically significant.

### Research ethics & compliance

This research was conducted in accordance with the ethical standards of the Declaration of Helsinki and was approved by the IRB of Sheikh Zayed Medical College & Hospital, Lahore (5s37-2025 IRB SZMC). All participants were made aware of the purpose and procedures of the study, risks and benefits and written or oral consent was obtained. Privacy and confidentiality were ensured by coded, secure data available to the research team. The participants were told that they could withdraw at any moment without reimbursement for their medical care.

## Results

### Demographic characteristics of study participants

The demographics of the 403 participants are summarised in Table 1. The age distribution was even: 18-25 years (21%), 26-30 years (24%), 31-35 years (25%), and 36-40 years (30%). Most participants were male (85%). The levels of education were not educated (27%), primary/secondary (38%), intermediate (28%), and university (7%). Students (23%), employed (34%), homemakers (29%), and unemployed (14%). The results of smoking were 24% never smokers, 32% former smokers, and 44% current smokers. The levels of physical activity were low (20%), moderate (49%), and high (31%). 65% of respondents reported having a family history of cardiovascular disease.

**Table 1:** Demographic characteristics of participants (N = 403)

Variable	f (N)	%		Variable	f (N)	%
Age				Occupation		
18-25 years	85	21		Student	93	23
26-30 years	95	24		Employed	139	34
31-35 years	100	25		Homemaker	115	29
36-40 years	123	30		Unemployed	56	14
Gender				Smoking status		
Male	342	85		Never smoked	96	24
Female	61	15		Former smoker	128	32
Educational level				Current smoker	179	44
No formal education	106	27		Physical activity level		
Primary/secondary school	155	38		Low (rarely active)	82	20
College/intermediate	112	28		Moderate (regular walking/light exercise)	196	49
University/graduate	30	7		High (frequent exercise/sports)	125	31
				Family history of cardiovascular disease		
				Yes	263	65
				No	140	35

Note. Values represent frequency (n) and percentage (%) of participants (N = 403)

### Spearman’s correlation among EHRA, AFEQT, and INTERHEART risk scores

Table 2 shows the Spearman correlations of the study variables. AFEQT showed a significant negative correlation with EHRA (p = -0.42, p < 0.001), and higher QoL was associated with greater symptom burden. EHRA was positively correlated with IHMRS (r = 0.13, p = 0.009), suggesting a slight increase in symptoms with increasing cardiovascular risk. IHMRS also showed a negative relationship with AFEQT (0.29, p < 0.001), indicating that worse QoL was associated with more advanced cardiovascular risk.

**Table 2:** Spearman's correlations among study variables (N = 403)

Variables	1	2	3
European heart rhythm association (EHRA)	-	ρ = -0.42, t(401) = -9.68, p < 0.001***	ρ = 0.13, t(401) = 2.63, p = 0.009**
Atrial fibrillation effect on quality-of-life (AFEQT)	-	-	ρ = -0.29, t(401) = -6.11, p < 0.001***
INTERHEART risk scores (IHMRS)	-	-	-

Note. Values are Spearman's rank-order correlation coefficients (ρ); p < 0.01 (**), p < 0.001 (***), N = 403; All percentages used in descriptive analysis are based on valid responses (i.e., excluding missing data) to ensure consistency across variables.

### Gender differences in EHRA, AFEQT, and INTERHEART risk scores (Mann-Whitney U test)

Table 3 provides gender-based comparisons using the Mann-Whitney U test. Women scored much higher on EHRA than men (mean ranks: 257.89 vs. 192.35; U = 7,221.00; p = 0.001), indicating a higher level of symptom burden. There were higher AFEQT scores in males (223.14 vs. 109.67; U = 5,024.00; p = 0.001), indicating better QoL. Male participants also exhibited higher IHMRS scores (210.76 vs. 122.43; U = 6,052.00; p < 0.001), indicating a higher cardiovascular risk. In general, there were considerable differences between genders in symptom severity, quality of life, and cardiovascular risk.

**Table 3:** Mann-Whitney U tests comparing European heart rhythm association (EHRA), atrial fibrillation effect on quality-of-life (AFEQT), and INTERHEART risk scores (IHMRS) by gender (N = 403)

Variable	Gender	n	Mean rank	Sum of ranks	U	Z	p
European heart rhythm association (EHRA)	Male	342	192.35	65,634.00	7,221.00	-3.42	0.001**
	Female	61	257.89	15,772.00			
Atrial fibrillation effect on quality-of-life (AFEQT)	Male	342	223.14	76,311.00	5,024.00	-6.02	< 0.001**
	Female	61	109.67	6,094.00			
INTERHEART risk scores (IHMRS)	Male	342	210.76	72,082.00	6,052.00	-5.48	< 0.001**
	Female	61	122.43	7,992.00			

Note. N = 403 (Males = 342, 85%; Females = 61, 15%); Mann-Whitney U test was used for all comparisons; p values marked with ** indicate statistical significance at p < 0.01, p < 0.001

### Age group differences in EHRA, AFEQT, and INTERHEART risk scores (Kruskal-Wallis Test)

Table 4 summarises Kruskal-Wallis results in four age groups. EHRA scores increased with age (mean ranks: 168.42, 195.76, 225.88, 262.41; χ^2^(3) = 29.54, p < 0.001), and older participants reported more symptoms. The mean AFEQT score across age levels (248.96 to 155.19) showed a reduction (χ^2^(3) = 34.17, p < 0.001; the lower the age, the worse the QoL), indicating that older people have worse quality of life. The INTERHEART Risk Scores also increased steadily (152.23 to 298.33; χ^2^(3) = 52.80, p < 0.001), reflecting an increase in cardiovascular risk in older cohorts. In general, there was a significant difference in symptom severity, QoL, and CVD risk across age groups.

**Table 4:** Kruskal-Wallis tests comparing total european heart rhythm association (EHRA), atrial fibrillation effect on quality-of-life (AFEQT), and INTERHEART risk scores (IHMRS) across age groups (N = 403)

Variable	Age group (years)	n	Mean rank	χ^2^ (df = 3)	p
European heart rhythm association (EHRA)	18-25 years	85	168.42	-	-
	26-30 years	95	195.76	-	-
	31-35 years	100	225.88	-	-
	36-40 years	123	262.41	29.54	< 0.001**
Atrial fibrillation effect on quality-of-life (AFEQT)	18-25 years	85	248.96	-	-
	26-30 years	95	210.37	-	-
	31-35 years	100	183.52	-	-
	36-40 years	123	155.19	34.17	< 0.001**
INTERHEART risk scores (IHMRS)	18-25 years	85	152.23	-	-
	26-30 years	95	201.6	-	-
	31-35 years	100	246.94	-	-
	36-40 years	123	298.33	52.80	< 0.001**

Note. N = number of participants in each age category; % = percentage of the total sample; Percentages are based on total N = 403; Values are mean ranks from Kruskal-Wallis H tests; Overall test statistics are reported in the bottom row for each variable: Total EHRA (χ^2^(3) = 29.54, < 0.001**), Total AFEQT (χ^2^(3) = 34.17, < 0.001**), and Total IHMRS (χ^2^(3) = 52.80, < 0.001**); Significance levels: p < 0.001**

### Multiple linear regression predicting INTERHEART modifiable risk scores

The results of the multiple linear regression of predictors of IHMRS are given in Table 5. Age was the best predictor of increased cardiovascular risk (β = 0.612, p < 0.001). Increased IHMRS were also independently associated with male sex (β = 0.104, p = 0.001), higher EHRA scores (β = 0.091, p = 0.001), and a family history of CVD (β = 0.079, p = 0.006). On the other hand, higher AFEQT scores (β = -0.188, p = 0.001) and greater physical activity (β = -0.109, p = 0.002) provided protection. The general findings were that the higher risk of CVD was predicted by older age, male gender, symptom burden, and family history and decreased by optimistic QoL and exercise.

**Table 5:** Multiple linear regression predicting INTERHEART modifiable risk score (IHMRS) from clinical and demographic factors (N = 403)

Predictor	B	SE	β	t	p	95% CI LL	95% CI UL
Constant (INTERHEART risk scores)	8.742	1.312	-	6.67	<0.001***	6.16	11.32
Total European heart rhythm association (EHRA)	0.682	0.198	0.091	3.44	0.001**	0.293	1.071
Total atrial fibrillation effect on quality-of-life (AFEQT)	-0.145	0.019	-0.188	-7.63	<0.001***	-0.182	-0.108
Age	3.214	0.242	0.612	13.29	<0.001***	2.738	3.690
Gender (1 = Male)	1.874	0.528	0.104	3.55	<0.001***	0.836	2.912
Physical activity	-0.698	0.218	-0.109	-3.20	0.002**	-1.126	-0.270
Family history of cardiovascular disease	0.854	0.310	0.079	2.76	0.006**	0.244	1.464

Note. N = 403, B = unstandardized regression coefficient; SE = standard error; β = standardised regression coefficient; CI = confidence interval; LL = lower limit; UL = upper limit; All predictors were entered; p < 0.01**, p < 0.001***.

### Significant predictors of INTERHEART risk scores

**Figure 1:**
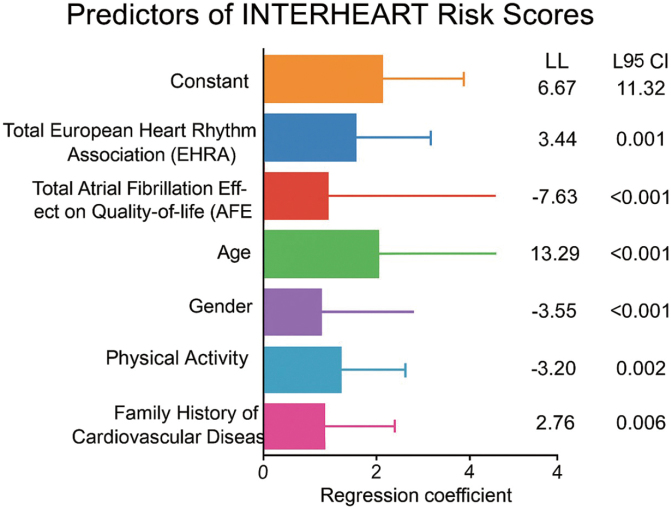
Significant predictors of INTERHEART risk scores in relation to cardiac risk and quality-of-life measures (N = 403). This figure was originally created by the authors

Figure 1 depicts the significant determinants of INTERHEART Risk Scores as well as cardiac risk and quality-of-life factors in 403 subjects. Regression analysis revealed that higher Total European Heart Rhythm Association (EHRA) scores and a positive family history of CVD were associated with higher INTERHEART risk scores, indicating greater cardiac risk. On the other hand, higher AFE scores, younger age, male sex, and higher levels of physical activity were associated with a lower INTERHEART risk score. Of these predictors, age was the strongest predictor of cardiac risk. At the same time, the AFE score significantly correlated inversely with it, indicating that both poor quality of life and older age dramatically increase cardiovascular risk.

## Discussion

The current study investigated the association between subclinical AF symptoms, quality of life (QoL), and cardiovascular risk in young adults using questionnaire-based assessments. In the present research, we also observed a significant negative association between symptom severity (EHRA) and quality of life (AFEQT), indicating that patients with the greatest AF-specific symptom severity tended to report poorer health-related quality of life. This is in line with prior reports and suggests that AF progression correlates with a deterioration of QoL, mainly driven by symptoms and adverse clinical events^15)^. In our study, a weak but significant relationship was observed between symptom severity (EHRA) and cardiovascular risk (IHMRS). This may imply that individuals with multiple cardiovascular risk factors perceive the symptoms of AF more severely than those without such factors. This observation is supported by a study that identified the same clinical risk factors as powerful predictors of AF development and progression^16)^. Furthermore, higher cardiovascular risk was also related to lower QoL, which is consistent with previous studies demonstrating that the presence of CVD risk factors is associated with worse quality of life in cardiac patients^17)^.

Females in our study had significantly higher EHRA scores and worse QoL than males, which was consistent with previous research showing that women suffer from more severe AF symptoms and poorer QoL compared to men^18, 19)^. On the other hand, men had higher CVD risk scores, consistent with international reports that traditional CVD risk factors are more often prevalent and associated with incidence of the disease in men^20)^.

In our analysis, a higher EHRA score was observed with increasing age, suggesting a tendency toward more severe symptoms with advancing age. This is in line with previous findings indicating that cardiac- metabolic and clinical risk factors contributing to AF become age-dependent and that older participants sustain a higher AF burden^21)^. In our research, the AFEQT slope decreases with age, and older ages are associated with lower quality of life. This is in line with previous studies that also showed age- related differences in AF-related QoL and symptom burden^22)^. In this study, cardiovascular risk identified by IHMRS increased significantly with age, suggesting a greater burden of risk factors among elderly participants. This is consistent with previous evidence that age is an independent determinant of cardiovascular disease, and the risk posed by frailty, obesity and diabetes accumulates^23)^.

In multivariable analysis, advanced EHRA score, ageing, and male sex were independent factors associated with higher cardiovascular risk, whereas higher AF-specific QoL was inversely related to this risk^16, 17, 20, 23)^. Family history of CVD was also an independent predictor, which is in accordance with previous studies demonstrating significantly increased risk in subjects with a positive family history of premature coronary artery disease^24)^. Furthermore, higher physical activity was inversely associated with cardiovascular risk, suggesting protection against cardiovascular disease through moderate exercise training. Evidence indicates that both physical activity and fitness are independently associated with decreased lipid abnormalities and clustered CVD risk factors^25)^.

Overall, these study findings support the value of using structured questionnaires to screen for subclinical AF and its associated cardiovascular risk indices among young adults at relatively low cost and in a scalable manner. Such initiatives can potentially facilitate targeted interventions in those with subclinical symptoms and high-risk profiles before irreversible cardiac remodelling occurs.

### Study constraints and recommendations

The present findings must be interpreted in light of several limitations. First, because of the cross-sectional design, no causal inferences can be drawn about the relationships among AF-related symptoms, quality of life, and cardiovascular risk. Second, the analysis was based solely on self-reported data collected through questionnaires, rather than on electrocardiographic (ECG) documentation of atrial fibrillation, which could have led to under- or overestimation of the prevalence of subclinical AF. Third, potential bias associated with convenience sampling might limit the generalisability of the results to the hospital population as a whole. Fourth, confounding factors including medication use, psychosocial stress and comorbidity were not adjusted in regression models. Third, cultural and linguistic factors may have influenced participants' comprehension of the questionnaire items, as no local translation or validation was provided.

Further studies using longitudinal cohorts that combine questionnaire-based screening with follow- up ECG recordings are needed to confirm the predictive value of self-reported AF symptoms for identifying true subclinical arrhythmias. Generalizability could be improved with a broader population-based or multi-regional study, which would allow investigation of demographic differences in risk. Furthermore, locally adapted translations of AF-related questionnaires need to be generated to enable appropriate context and translation. The addition of markers of inflammation, cardiac stress, or autonomic function might further elucidate mechanisms linking subclinical AF in young adults to cardiovascular risk. Lastly, intervention-based studies evaluating whether lifestyle modification, physical activity, or stress management can lower both AF-related symptom burden and cardiovascular risk would be of great benefit.

## Conclusion

The current analyses emphasise that the presence of a subclinical atrial fibrillation symptom in young adults, as identified by questionnaire, is robustly associated with worse cardiovascular health and health-related quality of life. Modifiable risk was significantly predicted by age, sex, family history and physical inactivity. These findings indicate the potential of using a questionnaire-based screening tool as an easy, low-cost means of early identification of those at risk in these resource-limited settings. Suppose we can add these screening tools to our usual health assessments. In that case, we might detect previously undiagnosed AF at a stage when preventive measures could be taken before it has any long-term consequences for the heart.

## Data availability

The datasets generated and analysed during the current study are available from the corresponding author on reasonable request.

## Author contributions

JAK and SA conceived and designed the study. BAB and HH contributed to data acquisition and verification. OJI and SP provided methodological input and conducted the literature review. KR and MV critically reviewed the manuscript for clinical relevance and intellectual content. SHK was responsible for referencing, formatting, and manuscript organisation. QM served as the corresponding author, coordinated the project, and finalised the manuscript. MM provided statistical guidance and public health contextualization. All authors read and approved the final version of the manuscript.

## Conflicts of interest statement

The authors declare that there are no conflicts of interest.
